# Processivity and enzymatic mechanism of a multifunctional family 5 endoglucanase from *Bacillus subtilis* BS-5 with potential applications in the saccharification of cellulosic substrates

**DOI:** 10.1186/s13068-018-1022-2

**Published:** 2018-01-29

**Authors:** Bin Wu, Shan Zheng, Marcelo Monteiro Pedroso, Luke W. Guddat, Siyuan Chang, Bingfang He, Gerhard Schenk

**Affiliations:** 1 College of Biotechnology and Pharmaceutical Engineering, Nanjing Tech University, 30 Puzhunan road, Nanjing, 211816 Jiangsu China; 2 China Jiangsu National Synergetic Innovation Center for Advanced Materials, 30 Puzhunan road, Nanjing, 211816 Jiangsu China; 3 School of Chemistry and Molecular Biosciences, The University of Queensland, St. Lucia, Brisbane, QLD 4072 Australia; 4 School of Pharmaceutical Sciences, Nanjing Tech University, 30 Puzhunan road, Nanjing, 211816 Jiangsu China

**Keywords:** *Bacillus subtilis*, Processive endoglucanase, Family 5 glycoside hydrolase, Multifunctional, Saccharification

## Abstract

**Background:**

Presently, enzymes still constitute a major part of the cost of biofuel production from lignocellulosic biomass. Processive endoglucanases, which possess both endoglucanase and exoglucanase activity, have the potential to reduce the costs of biomass saccharification when used together with commercial cellulases. Therefore, the exploration of new processive endoglucanases has attracted much attention with a view to accelerating the industrialization of biofuels and biochemicals.

**Results:**

The endoglucanase EG5C and its truncated form EG5C-1 from *Bacillus subtilis* BS-5 were expressed and characterized. EG5C was a typical endoglucanase, comprised of a family 5 catalytic domain and family 3 carbohydrate-binding domain, and which had high activity toward soluble cellulosic substrates, but low activity toward insoluble cellulosic substrates. Importantly, the truncated form EG5C-1 was a processive endoglucanase that hydrolyzed not only carboxymethyl cellulose (CMC), but also insoluble cellulosic substrates. The hydrolytic activities of EG5C-1 towards CMC, phosphoric acid-swollen cellulose (PASC), *p*-nitrophenyl-β-d-cellobioside, filter paper and Avicel are 4170, 700, 2550, 405 and 320 U/μmol, respectively. These data demonstrated that EG5C-1 had higher activity ratio of exoglucanase to endoglucanase than other known processive endoglucanases. When PASC was degraded by EG5C-1, the ratio of soluble to insoluble reducing sugars was about 3.7 after 3 h of incubation with cellobiose and cellotriose as the main products. Importantly, EG5C-1 alone was able to hydrolyze filter paper and PASC. At 5% substrate concentration and 10 FPU/g PASC enzyme loading, the saccharification yield was 76.5% after 60 h of incubation. Replacement of a phenylalanine residue (F238) by an alanine at the entrance/exit of the substrate binding cleft significantly reduces the ability of EG5C-1 to degrade filter paper and Avicel, but this mutation has little impact on CMCase activity. The processivity of this mutant was also greatly reduced while its cellulose binding ability was markedly enhanced.

**Conclusions:**

The processive endoglucanase EG5C-1 from *B. subtilis* BS-5 exhibits excellent properties that render it a suitable candidate for use in biofuel and biochemical production from lignocellulosic biomass. In addition, our studies also provide useful information for research on enzyme processivity at the molecular level.

**Electronic supplementary material:**

The online version of this article (10.1186/s13068-018-1022-2) contains supplementary material, which is available to authorized users.

## Background

Cellulose, the most abundant renewable feedstock for biofuels and chemicals production, consists of linear chains of β-1,4-linked glucose units that form a higher order crystalline structure [1]. Cellulolytic microorganisms, including fungi and bacteria, are highly proficient in degrading cellulose into fermentable sugars. Most aerobic fungi and bacteria secret a series of free cellulases, known as the noncomplexed cellulase system, to degrade cellulose [2]. An alternative mechanism for the bioconversion of cellulose was found in quite a few anaerobic microorganisms such as *Clostridium thermocellum,* which employs a large multiprotein complex (cellulosome) to hydrolyze cellulose [3]. In both strategies, i.e. aerobic and anaerobic, the efficient hydrolysis of cellulose to glucose depends on the synchronized action of three classes of enzymes, endoglucanase, exoglucanase and β-glucosidase. Processivity is a common mode of action for many exoglucanases and plays a key role in the complete hydrolysis of crystalline cellulose. In contrast, classic endoglucanases randomly cleave β-1,4-glycosidic bonds in the interior of the cellulosic chains and have been considered to be non-processive [4]. Recently, a few microorganisms such as *Cytophaga hutchinsonii* and *Saccharophagus degradans* were found to be able to rapidly digest cellulose [5, 6]. However, a bioinformatics analysis indicates that the genomes of these organisms did not possess any apparent homologues to known exoglucanases [5, 6]. Furthermore, several endoglucanases that possess both endo and exoglucanase activity and appear to act in processive manner were found in these organisms, potentially presenting a novel strategy for the degradation of cellulose [5–7]. Here, processive manner (processivity) can be defined as continuous threading of a single carbohydrate chain through the catalytic site of the enzyme, yielding oligosaccharide in successive cleavages [8]. In the commercialization of cellulose biorefineries, the cost of enzyme cocktails is a major economic barrier [9]. Therefore, in contrast to other cellulolytic systems that dependent on both endo and exoglucanases, a processive endoglucanase coupled with a β-glucosidase may be sufficient for the degradation of cellulose and consequently this enzyme has attracted considerable interest [6].

Presently, most of the known processive endoglucanases belong to GH9 family, which displays a modular structure with a catalytic domain from family 9 and various carbohydrate-binding modules (CBMs) [6]. These CBMs are to be critical for the processivity of GH9 endoglucanases. Without CBMs, these enzymes act more like classical endoglucanases [10–12]. Only a few processive endoglucanases from the GH5 family have been identified to date. Their mechanism for enzymatic processivity appears to be more complicated than that of the GH9 family. For example, processive endoglucanase EG1 from *Volvariella volvacea* consists of the N-terminal CBM1 and the C-terminal GH5 catalytic domain. Deletion of CBM1 domain significantly reduces the ratio of soluble to insoluble products from filter paper, supporting a critical role of CBM1 for EG1 processivity [13]. In contrast, the processivity of another GH5 endoglucanase Cel5H from *S. degradans* 2–40 is not affected by the absence/presence of the CBM6 module. It is thus speculated that in this enzyme the catalytic domain alone is essential for the processivity mechanism [6]. Further, two GH5 endoglucanases Cel5A and CHU_2103 without CBM have been reported successively [14, 15]. A site-directed mutagenesis study of CHU_2103 from *C. hutchinsonii*, has identified the aromatic amino acid W197 as essential for processivity [15], but the overall mechanism remains obscure.

Until recently, most commercial lignocellulolytic enzymes were produced by filamentous fungi. However, the use of bacteria as highly potent sources for cellulase production has gained increasing attention due to the more complex glycoside hydrolases in these organisms, great natural diversity and higher growth rate [16]. In our previous study, strain *B. subtilis* BS-5 with a complete cellulolytic system was isolated and characterized; this system was shown to rapidly and efficiently degrade cellulose [17]. However, *Bacillus* species commonly lack significant exoglucanase activity. Indeed, an analysis of the genome sequence of *B. subtilis* 168 indicated that the lignocellulolytic system of this strain contained an endoglucanase, two xylanases and five β-glucosidases, but no genes encoding proteins homologous to any known family of exoglucanases has been found [18]. The mechanism employed *B. subtilis* BS-5 to degrade cellulose thus remains unknown. We hypothesized that *B. subtilis* BS-5 might produce a processive endoglucanase, which might substitute for the apparent deficiency in exoglucanase activity. However, to the best of our knowledge, no processive endoglucanase from *B. subtilis* has yet been reported. To substantiate our hypothesis, we purified and characterized three secreted proteins from *B. subtilis* BS-5 using a cellulose-binding strategy. A multidomain enzyme, EG5C, containing a family 5 catalytic domain and a family 3 CBM, and its two truncated forms, EG5C-1 (comprising the catalytic domain only) and EG5C-2 (translocating CBM3 domain from C-terminus of EG5C to its N-terminus), were expressed in *E. coli*, and characterized to analyze the contribution of the CBM to the catalytic activity and mode of action of these enzymes. In addition, mutants of EG5C-1 were generated to probe its processivity mechanism and cellulose binding ability. Finally, the potential of EG5C-1 for applications in saccharification of cellulosic substrates was evaluated.

## Methods

### Strains, plasmids and culture conditions

*Bacillus subtilis* BS-5 was isolated by combined carboxymethyl cellulose agar plate, xylan agar plate and filter paper hydrolysis assays, and cultivation was performed as previously described [17]. *E. coli* DH5α was used for plasmid constructions and propagation. pET 28a (Novagen) and *E. coli* BL21 were used as the host and vector for the expression of recombinant enzymes. Bacterial strains were grown at 37 °C in Luria-Bertani (LB) medium, supplemented with 100 μg/mL ampicillin.

### Purification and identification of native enzymes EG5C and EG5C-1

Fermentative supernatant of *B. subtilis* BS-5 was mixed with Avicel at a final concentration of 10% (w/v) at 4 °C under gentle shaking for 4 h. Subsequently, the mixture was centrifuged to separate the cellulosic substrate. The pelleted fraction was washed several times with distilled water to limit nonspecific protein binding. Bound proteins were eluted with SDS-PAGE buffer, and subjected to SDS-PAGE analysis on a 12% gel. The Coomassie-stained bands of the bound proteins were excised from the gel for LC/MS–MS analysis, and the sequences of the fragments were submitted to the Mascot program for possible identity matching.

### Construction of plasmids and site-directed mutagenesis

The DNA sequence encoding the mature endoglucanase BsCel5A (NCBI No. NP389695.2) of *B. subtilis* 168 was used to design the primer pairs to amplify the genes encoding EG5C and EG5C-1 [19]. For EG5C, the primers, EG5C-F and EG5C-R, were used. The truncated form EG5C-1, lacking the CBM module, was amplified using EG5C-1-F and EG5C-1-R as forward and reverse primers, respectively (Additional file 1: Table S1). The amplified DNA fragments were digested with *Nco* I and *Xho* I, and then ligated into a similarly treated pET 28a vector to generate the expression vectors pET-EG5C and pET-EG5C-1. The variant EG5C-2 was formed by the translocation of CBM3 domain from C-terminus of EG5C to its N-terminus. Therefore, the pET-EG5C-2 system was constructed by inserting a fragment consisting of CBM3 and a 22-amino acid linker (TKDSTKDIPETPSKDSHTQENG) upstream of the catalytic domain of EG5C-1. For this purpose, the fragment encoding CBM3, the linker and the catalytic domain was amplified by PCR using the primer pair EG5C-2-F and EG5C-2-CBM-R, the primer pair EG5C-2-LIN-F and EG5C-2-LIN-R, the primer pair EG5C-2-GH-F and EG5C-2-R, respectively (Additional file 1: Table S1). Subsequently, the fragments encoding CBM3, the linker and the catalytic domain were amplified by overlapping PCR using the primer pair EG5C-2-F and EG5C-2-R. CBM3, linker and catalytic domain fragments were used as template. PCR amplification was performed in a final reaction volume of 20 μL containing 10 μL of 2× ExTaq (TaKaRa) mix reaction buffer, 100 ng of template genome DNA and 2 μL of 0.2 μmmol/L each of forward and reverse primers. For EG5C and EG5C-1, the thermal cycling conditions included an initial denaturation at 94 °C for 5 min followed by 30 cycles of 94 °C for 30 s, 55 °C for 30 s and 72 °C for 2 min. The overlapping PCR cycling conditions were 10 of above cycles without primers, and then 20 of the above cycles after adding primers. Finally, the amplified fragment was inserted into pET28a to generate expression vector pET-EG5C-2. All recombinant plasmids containing pET-EG5C, pET-EG5C-1 and pET-EG5C-2 were transformed into *E. coli* DH5α for plasmid DNA propagation. After the correct construction was confirmed by DNA sequencing, these recombinant plasmids were transformed into *E. coli* BL21 for protein expression. All engineered EG5C variants were fused to a hexa-histidine tag at the C-terminus to facilitate easy purification via affinity chromatography.

Site-directed mutagenesis of EG5C-1 was carried out by overlap extension PCR and the required primers were listed in Additional file 1: Table S2. All mutated plasmids were verified by sequencing and then transformed into *E. coli* BL21 to produce recombinant mutant proteins. These point mutations were purified and characterized following the procedures described above.

### Expression and purification of enzymes

Recombinant *E. coli* BL21 cells harboring the plasmids described above were cultured in LB medium and the target genes were expressed as described above [5, 15]. After expression, the cells were harvested by centrifugation and disrupted by sonication. Recombinant proteins were purified by affinity chromatography with a HisTrap FF column (GE Healthcare, Fairfield, USA) according to the manufacturer’s recommendations. Enzyme homogeneity and the molecular weights of purified EG5C, EG5C-1 and EG5C-2 were estimated on a 12% SDS-PAGE gel. The protein concentrations were measured by the method of Bradford [20].

### Enzyme activity assays

The hydrolytic activity of EG5C and its derivatives were measured in 2 mL of a reaction mixture containing 2.5% of the desired substrates (w/v, 1.5 mL) in 50 mmol/L Na_2_HPO_4_/KH_2_PO_4_ buffer (pH 6.5) and 0.5 mL of suitably diluted enzyme at 45 °C [17]. The polysaccharide substrates used in this study included carboxymethyl cellulose (CMC), β-glucan, xylan, Avicel, phosphoric acid-swollen cellulose (PASC), Whatman filter paper (FP) and chitosan. For assays with CMC, β-glucan and xylan, the reaction duration was 30 min, while a mixture of substrate and enzyme was incubated for 1 h in the assays with Avicel, PASC, FP and chitosan. PASC was prepared as described previously [15]. After incubation in water bath with shaking for 30 min or 60 min, the amount of reducing sugar released was determined using the 3,5-dinitrosalicylic acid method [17]. One unit of enzymatic activity was defined as the amount of enzyme that liberated 1 μmol of reducing sugar equivalents per minute. The hydrolysis of *p*NPC (10 mmol/L) was assayed by monitoring the concentration of released *p*-nitrophenol at 410 nm after addition of NaOH at a final concentration of 0.1 mmol/L as described by Ghatge [21].

For viscosity measuring, the reaction was performed at 45 °C in 15 mL total volume containing purified enzyme (15 μg) and 2.5% CMC solution at pH 6.5. Then, samples were taken periodically and boiled for 5 min, and viscosity values were determined using an Ubbelohde viscometer tube (Fisher Scientific, Waltham, US).

### Biochemical characterization of E5GC and its derivatives

The effects of pH and temperature on the activity of EG5C and its derivatives were measured using CMC as substrate by changing the reaction pH value from 3 to 10 at 45 °C and temperature from 30 to 80 °C at a pH of 6.5, respectively. Thermal stability was estimated by measuring the residual activities after 2 h incubation at a specific temperature ranging from 30 to 80 °C with temperature increments of 10 °C. Enzyme activity prior to incubation was expressed as 100%. pH stability was evaluated by measuring the residual activities after 2 h of incubation in different buffers with pH values ranging from 3 to 9 at 45 °C.

Kinetic constants (*K*_m_, *V*_max_) were determined using CMC (at concentrations from 1 to 20 mg/mL), Avicel and PASC (ranging from 1 to 200 mg/mL) as substrates under optimum reaction conditions, respectively. The data were analyzed using nonlinear regression with GraphPad Prism 5.0 software (http://www.graphpad.com/prism/). Turnover rate (*k*_cat_) and catalytic efficiency ratio (*k*_cat_*/K*_m_) were calculated from the obtained kinetic data.

### Analysis of hydrolysis products by thin-layer chromatography

Hydrolysis of CMC, Avicel and PASC by EG5C and its derivatives was performed for 6 h under the same conditions used for the activity assays and terminated by heating the samples in boiling water bath for 10 min. After centrifugation at 10,000×*g* for 10 min, the samples were spotted on silica 60F_254_ TLC plates and air dried. Thin-layer chromatography (TLC) was performed using a mixture of *n*-butanol, acetic acid and water in a volume ratio of 3:2:1. Then, the hydrolysis products were visualized by spraying the plates with an ethanol/sulfuric acid mixture (90:10, v/v) and heating at 120 °C for 10 min. Hydrolysis of cello-oligosaccharides with the enzymes was carried out for 1 h and analyzed according to the method described above.

### Assay of cellulose-binding capacity

The binding capacity of EG5C and its derivatives to the cellulosic substrate Avicel was assayed in Eppendorf tubes containing recombinant protein and substrate as described by Zhang et al. [15]. The concentrations of Avicel and the recombinant protein were 50 mg/mL and 0.5 mg/mL in a final volume of 1.0 mL of 20 mmol/L Na_2_HPO_4_/KH_2_PO_4_ buffer (pH 6.5). After incubation at 4 °C for 1 h, the samples were centrifuged (10,000×*g*) to remove Avicel and the bound protein. The concentration of unbound protein remaining in the supernatant was measured by the method of Bradford [20]. Bound protein was removed from Avicel using the sample buffer of SDS-PAGE as described by Ghatge et al. [21]. The initial, unbound and released proteins were analyzed by 12% (w/v) SDS-PAGE.

### Processivity assay

Processivity of EG5C and its derivatives was determined by measuring the ratio of soluble to insoluble reducing sugar from PASC [7, 15]. In brief, the reaction with 0.5% of PASC and 50 μg/mL of recombinant protein was carried out at 45 °C, and a sample was removed from the mixture at different time points. After separation by centrifugation, the amount of released reducing sugars in the supernatant and in the remaining PASC fraction was determined by the 3,5-dinitrosalicylic acid method [17].

### Degradation of filter paper and PASC by EG5C-1

With filter paper, the hydrolysis reactions were carried out at a 2.5% (w/v) solid loading in Na_2_HPO_4_/KH_2_PO_4_ buffer (20 mmol/L, pH 6.5) at a final volume of 20 mL. EG5C and EG5C-1 were added to the reaction mixture at a concentration of 40 μg/mL for each. The mixture was shaken at 200 rpm at 40 °C for 24 h. The control assay without enzyme was conducted under the same conditions.

The hydrolysis of PASC was performed in a 100-mL Erlenmeyer flask and in Na_2_HPO_4_/KH_2_PO_4_ buffer (20 mmol/L, pH 6.5) with 5% (w/v) of PASC and 10 FPU/g PASC of EG5C-1. The reaction mixture (20 mL) was incubated in a water bath at 40 °C for 60 h. Samples from the reaction were collected every 12 h and quantified by high performance liquid chromatography (HPLC), equipped with a UltiMate 3000 pump (Dionex, USA) and a refractive index detector (R1-101, Dionex). A 100-5 NH_2_ column (4.6 × 250 mm, Kromasil) was used for HPLC analysis. The mobile phase comprised acetonitrile and water in a ratio of 75:25 and at a flow rate of 0.7 mL/min. Synergistic hydrolysis by the commercial β-glucosidase (Novozyme 188, Novozymes) and EG5C-1 under the same conditions was implemented as a control. The loading rates of β-glucosidase and EG5C-1 were 20 U/g PASC and 10 FPU/g PASC, respectively. The hydrolysis yield was defined as the ratio of the total equivalents of glucan hydrolyzed to the total potential glucan available in PASC.

### Computational analysis

Nucleotide and amino acid sequences were analyzed using the BLASTn and BLASTp programs. The signal peptide of EG5C was predicted using SignalP 4.0 web program (http://www.cbs.dtu.dk/services/SingnalP/). Multiple sequence alignments were carried out using Clustal Omega program suite (http://www.ebi.ac.uk/Tools/msa/clustalo/). Homology modeling of EG5C-1 was performed with Discovery studio (DS) 3.5 MODELER based on the template of the crystal structure of the catalytic domain of BsCel5A from *B. subtilis* 168 [19]. An automated feature-based docking of substrates, i.e. cellotriose and cellotetraose in the catalytic sites of EG5C-1 was performed using the DS 3.5 docking program combined with CHARMM [21].

## Results and discussion

### Purification and identification of enzymes EG5C and EG5C-1

**Fig. 1:**
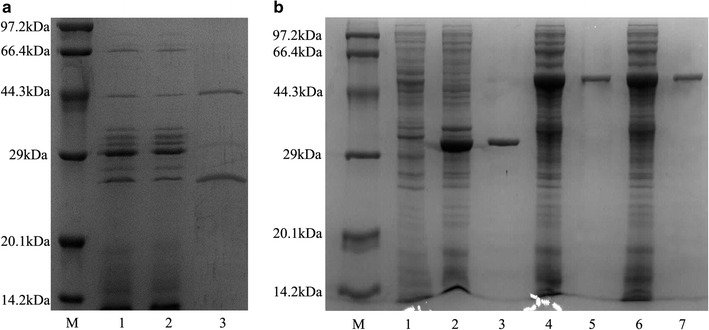
**a** SDS-PAGE analysis of cellulose-binding proteins secreted by *B. subtilis* BS-5. Lane M marker, Lane 1 fermentation broth of *B. subtilis* BS-5, Lane 2 unbound fractions in the fermentation broth after Avicel adsorption, Lane 3 cellulose-binding proteins bound to Avicel, eluted with SDS-PAGE buffer. **b** SDS-PAGE analysis of recombinant EG5C, EG5C-1 and EG5C-2 produced by *E. coli*. Lane M-marker, Lane 1-crude extracts of BL21-pET28a, Lanes 2, 4 and 6-crude extracts of E-pET-*eg5c*-*1*, E-pET-*eg5c*, E-pET-*eg5c*-*2*, Lines 3, 5 and 7-purified EG5C-1, EG5C and EG5C-2 with a HisTrap FF column

A purification strategy based on the high affinity of cellulolytic enzymes to cellulose was used to isolate proteins secreted by *B. subtilis* BS-5. An SDS-PAGE analysis revealed that three proteins were bound to Avicel with apparent molecular weights of 46, 33 and 27 kDa, respectively (Fig. 1a). Then, these proteins were excised from the gel and the corresponding proteins were identified by trypsin digestion and partial amino acid sequencing. A search with selected mass values against the Mascot Peptide Mass Fingerprint data bank indicated that the fragments associated with the 46 kDa protein matched those of the endoglucanase from *B. subtilis* 168 (NCBI Accession No. NP 389695) [19]. The fragments obtained from 27 kDa band showed significant homology to the protein expansion YoaJ from *B. subtilis* 168 (NCBI Accession No. NP 389744.1). Surprisingly, the fragments linked to the 33 kDa protein also displayed high homology to the endoglucanase of *B. subtilis* 168. However, the matched peptides only appeared in the catalytic domain of this enzyme (Additional file 1: Fig. S1). Therefore, we concluded that the 33 kDa enzyme observed on the SDS-PAGE gel represented a truncated form of the 46 kDa enzyme which might be produced by limited proteolysis with a protease from strain BS-5.

The gene encoding the 46 kDa enzyme had an open reading frame of 1500 bp translating into a protein of 499 amino acids with a signal peptide of 29 amino acids, predicted using the SignalP 4.0 program. A domain analysis indicated that the mature 46 kDa enzyme (i.e. EG5C) was composed of a glycoside hydrolase family 5 catalytic domain of 302 amino acids, a linker region of 22 amino acids and a CBM3a module of 146 amino acids (Additional file 1: Fig. S1). The 33 kDa enzyme (labeled EG5C-1) only contained the catalytic domain of EG5C (Additional file 1: Fig. S1). A multiple sequence alignment of EG5C with other known GH5 family endoglucanases revealed a high degree of sequence identity with typical endoglucanases from *B. subtilis* 168, *B. subtilis* A53, *B. amyoliquefaciens* DL-3 and *B. subtilis* UMC7 (Additional file 1: Fig. S2) [19, 22–24]. While the enzymatic properties and function of these enzymes have been well understood, the roles and the synergy between their catalytic and CBM domains remain unclear.

### Expression, purification and characterization of EG5C and its derivatives

To investigate biochemical properties and role of EG5C and its C-terminal CBM3 module in the hydrolysis of cellulose, EG5C and its derivatives were successfully expressed in *E. coli*. The variants were purified by affinity chromatography using HisTrap FF column. An SDS-PAGE analysis indicated that all enzymes migrated as single dominant band with expected molecular weights (Fig. 1b). The specific activities of EG5C, EG5C-1 and EG5C-2 towards CMC were 5310, 4170 and 3820 U/μmol, respectively.

**Fig. 2:**
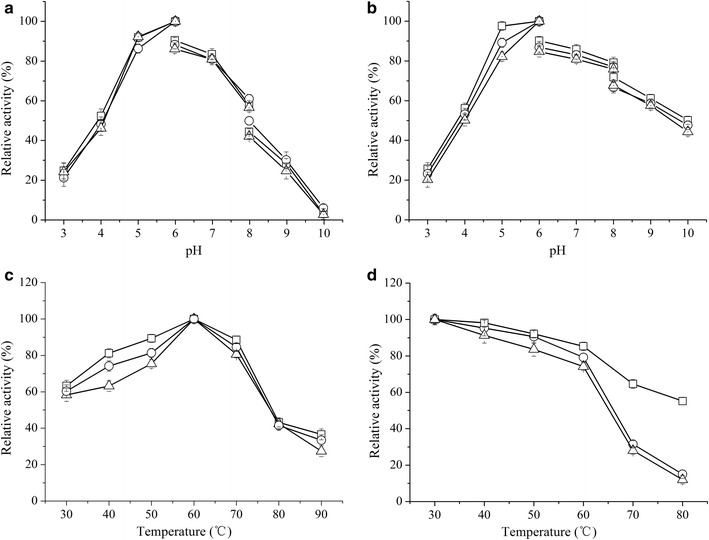
Effect of temperature and pH on the activity and stability of EG5C and its derivatives. **a** Activity of EG5C (○), EG5C-1 (□) and EG5C-2 (∆) was measured at different pH values at 45 °C. **b** pH stability of EG5C (○), EG5C-1 (□) and EG5C-2 (∆) evaluated following incubation at 45 °C for 2 h at different pH values. The buffer systems were as follows: 50 mmol/L citric acid/sodium citrate (pH 3.0–6.0), 50 mmol/L Na_2_HPO_4_/KH_2_PO_4_ (pH 6.0–8.5), 50 mmol/L Gly/NaOH (pH 8.5–10.5). **c** The effect of temperature on activity of EG5C (○), EG5C-1 (□) and EG5C-2 (∆) was studied by changing the temperature from 30 to 80 °C at pH 6.5. **d** Thermosatbility of EG5C (○), EG5C-1 (□) and EG5C-2 (∆) measured by incubating the enzyme at various temperatures for 2 h. Error bars are standard deviation of 3 independent experiments

The effects of pH and temperature on activity and stability of EG5C and its derivatives were analyzed using CMC as substrate (Fig. 2). Maximum activity of EG5C was observed at pH 6.0 and a temperature of 60 °C. Similar pH and temperature optima have been previously been reported for endoglucanases produced by *B. subtilis* UMC7, *B. subtilis* JA18 and *B. subtilis* LH [24–26], while the optimum temperature of endoglucanases produced by *B. amyloliquefaciens* DL-3 and *B. subtilis* A-53 was slightly lower at 50 °C [22, 23]. The derivatives EG5C-1 and EG5C-2 also had similar optimal pH and temperature (Fig. 2a). Moreover, EG5C and its derivatives had similar stability over a broad pH range from 3 to 10, retaining more than 80% of the initial activity after incubation for 2 h at various pH values (Fig. 2b). These results indicated that the removal of CBM3 had no effect on the optimal pH and temperature of EG5C, which was in agreement with previous reports on similar enzymes [25, 26].

While the three variants had a similar temperature optimum (Fig. 2c), they differed with respect to their thermal stability. EG5C and EG5C-2 retained only about 15% of their original activities after 2 h of incubation at 80 °C. In contrast, EG5C-1 was significantly more stable with nearly 60% of its original activity still present after 2 h incubation at 80 °C (Fig. 2d). Thus, the deletion of CBM3 significantly improved the thermal stability of endoglucanase EG5C. Most previous studies indicated that the removal of CBM domain from endoglucanase was deleterious to its thermal stability. For instance, in a study by Zhang et al. the deletion of CBM3 from Ba-EGA produced by *Bacillus* sp. AC1 reduced the thermal stability of this endoglucanase [27]. Chiriac et al. extracted the family 9 endoglucanase Cel9B from *P. barcinonesis* which contained two CBMs at the C-terminus [10]. A series of truncated derivatives of Cel9B was constructed and characterized, and the truncated forms devoid of CBM3c were completely inactivated after incubation for 30 min at 30 °C, while partially truncated forms that still contained CBM3c remained stable after 1 h at 50 °C [10]. Furthermore, Pan et al. reported that fusion of an extra CBM1 domain with the non-modular family 5 endoglucanase FmEG∆24 had higher thermal stability [28]. To date only a few studies showed that deletion of a CBM domain could enhance the thermal stability of an intact endoglucanase. Wang et al. reported a great improvement in thermal stability upon truncating the C-terminus of the endoglucanase Egl499 [25]. Another endoglucanase with enhanced thermostability in the truncated, CBM-free form was BsCel5A from *B. subtilis* 168, here, the reduced stability of the intact (full-length) enzyme was ascribed to the low stability of CBM3 in comparison to the catalytic domain [18]. Due to the high sequence identity between endoglucanases EG5C and BsCel5A (99%), it was likely that the relative instability of the CBM3 domain in EG5C was the main factor leading to the increased thermostability of the truncated EG5C-1 variant. Since enhanced thermostability was a critical, cost-reducing property of enzymes in industrial applications, EG5C-1 may be a promising candidate for processes requiring the saccharification of lignocellulose [19, 25].

### Cellulolytic activity and processivity of EG5C and its derivatives

**Fig. 3:**
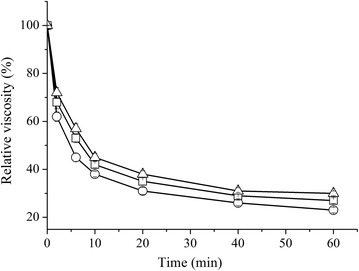
Effect of EG5C (○), EG5C-1 (□) and EG5C-2 (∆) on the viscosity of a CMC solution. Error bars are standard deviation of 3 independent experiments

In order to assess the catalytic potential of EG5C and its derivatives, their activities towards a range of substrates were tested. As shown in Fig. 3, EG5C and its derivatives EG5C-1 and EG5C-2 were able to decrease the viscosity of CMC by nearly 70% after 60 min of incubation, indicating an endo-action hydrolytic mode [7, 26]. Specific activities for a range of cellulosic substrates were summarized in Table 1. EG5C had the highest activity towards β-glucan (7890 U/μmol), followed by CMC (5310 U/μmol) and *p*-NPC (330 U/μmol). With respect to insoluble cellulose substrates, EG5C was able to hydrolyze PASC (110 U/μmol), but its activity towards other insoluble substrates was either very low (FP and Avicel) or non-existent (xylan and chitosan). These trends and observations were in agreement with those reported for typical endoglucanases from *B. subtilis* 168, *B. subtilis* LH and *B. subtilis* UMC7 [18, 24, 26]. The translocation of the CBM3 domain from the C-terminus of EG5C to its N-terminus (forming variant EG5C-2) had no effect on the substrate specificity, but the cellulolytic activities were lower than those of native EG5C (Table 1). In contrast, the truncated, CBM-free EG5C-1 displayed notable differences in substrate preference. While its activity for CMC and β-glucan was somewhat lower than that of the native enzyme, this variant had a strongly enhanced activity for insoluble substrates tested and *p*NPC (no activity was detected for xylan or chitosan).

**Table 1:** Substrate specificity of EG5C and its derivatives

Substrate	Specific activity (U/μmol)		
	EG5C	EG5C-1	EG5C-2
CMC	5310	4170	3820
β-glucan	7890	2790	5390
PASC	110	700	70
*p*NPC	330	2550	245
Filter paper	10	405	5.2
Avicel	5.5	320	3.5
Xylan	0	0	0
Chitosan	0	0	0

Enzyme reactions were conducted at 45 °C and pH 6.5. For assays with CMC, β-glucan and xylan, the reaction duration was 30 min, while a mixture of substrate and enzyme was incubated for 1 h in the assays with Avicel, PASC, FP and chitosan. The hydrolysis of *p*NPC (10 mmol/L) was assayed by monitoring the concentration of released *p*-nitrophenol at 410 nm after addition of NaOH at a final concentration of 0.1 mmol/L

The catalytic parameters recorded for the reactions with CMC, PASC and Avicel were summarized in Table 2. When CMC was used as the substrate, the *K*_m_, *k*_cat_ and *k*_cat_*/K*_m_ of EG5C were 5.7 mg/mL, 218/s and 38 mL/mg/s, respectively, while these values for EG5C-1 were 6.5 mg/mL, 193/s and 30 mL/mg/s. From a comparison between EG5C and EG5C-1, it was apparent that the CBM3 domain contributed to a moderate enhancement of substrate affinity (estimated from *K*_m_ values) and catalytic efficiency (determined from *k*_cat_/*K*_m_ ratio) in the reaction with CMC. However, only the truncated variant retained significant catalytic activity towards the insoluble polysaccharides PASC and Avicel. Usually, Avicel and *p*-NPC are used as substrates to measure the activity of exoglucanases as they are not commonly hydrolyzed by endoglucanases [29]. The data presented here demonstrated, however, that EG5C-1 acted as a processive endoglucanase on insoluble substrates, exhibiting both “endo” and “exo” types of activity. The Avicelase and FPase activities of EG5C-1 were 320 and 405 U/μmol, which reached 7.6 and 9.7% of its CMCase activity. Importantly, while some family 9 and family 5 processive endoglucanases from fungi and bacteria have been reported [6, 10, 11, 13, 21, 30], EG5C-1 exhibited higher activity ratio of exoglucanase to endoglucanase. Consequently, EG5C-1 is a highly promising candidate enzyme to reduce costs of efficient lignocellulose bioconversion.

**Table 2:** Kinetic parameters of EG5C and its derivatives at optimal reaction condition

Substrate	EG5C			EG5C-1			EG5C-2		
	*K*_m_ (mg/mL)	*k*_cat_ (/s)	*k*_cat_*/K*_m_ (mL/mg/s)	*K*_m_ (mg/mL)	*k*_cat_ (/s)	*k*_cat_*/K*_m_ (mL/mg/s)	*K*_m_ (mg/mL)	*k*_cat_ (/s)	*k*_cat_*/K*_m_ (mL/mg/s)
CMC	5.7	218	38	6.5	193	30	5.9	145	25
PASC	N	N	N	22.7	41.3	1.6	N	N	N
Avicel	N	N	N	74.6	2.4	0.1	N	N	N

N means not detected. Kinetic study was performed at 60 °C and pH 6.0. The data were analyzed using non-linear regression with GraphPad Prism 5.0 software

**Fig. 4:**
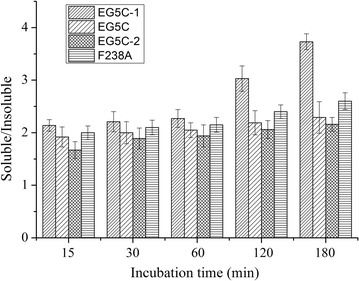
The ratios of the soluble to insoluble reducing sugars generated by EG5C, EG5C-1, EG5C-2 and F238A mutant of EG5C-1, respectively. Error bars are standard deviation of 3 independent experiments

The processivity of endoglucanases has been mainly determined from the ratio of soluble *vs* insoluble reducing ends generated from cellulosic substrates [6, 21]. In this study, the distribution of reducing sugars generated by EG5C, EG5C-1 and EG5C-2 was measured using PASC as substrate (Fig. 4). For EG5C and EG5C-2, the ratios of soluble to insoluble fractions increased slightly from 1.92 to 2.29 and 1.67 to 2.16 as the incubation time was prolonged from 15 min to 180 min. In contrast, more soluble reducing sugars were produced by EG5C-1, especially as the incubation period was increased (from 2.14 after 15 min to 3.73 after 180 min). For comparison, the ratio increased from 2.69 to 3.72 for the processive endoglucanase CHU_2103 when studied under similar conditions [15]. Furthermore, for two processive endoglucanases Cel9A and MtEG5, the ratios of soluble to insoluble products using FP as a substrate were 4.26 after 2 h of incubation and 3.91 after 1 h of incubation, respectively [7, 12]. And for the processive endoglucanase AS-HT-CeluzA from *A. ochraceus* MTCC1810, the processivity ratio increased from 1.79 to 3.1 when the incubation time was prolonged from 3 h to 9 h [12]. Although a higher ratio of soluble *vs* insoluble products was observed for endoglucanase EG1 (8.6), the incubation time was 24 h [13]. Generally, for the hydrolysis of insoluble substrates, typical endoglucanases produce 30–60% of soluble reducing sugars, while the yield is as high as 90% for exoglucanases [15]. For EG5C and EG5C-1, the yield was 69.6 and 78.9% of soluble reducing sugars from PASC, respectively, demonstrating processivity enhancement of EG5C upon the deletion of the CBM domain.

Since processivity is critical for cellulases to catalyze the degradation of crystalline cellulose, exoglucanases have been playing a key role in the solubilization of cellulose in both the complexed and non-complexed cellulolytic systems [31, 32]. The processive mechanism of exoglucanases has been investigated in great detail [33]. The corresponding mechanism employed by processive endoglucanases is far more obscure. It is known that the function of CBM domains is to recognize and bind specifically to insoluble cellulose, thereby increasing the affinity and activity of the cellulases [8, 34]. For most, but not all, endoglucanases processivity also depend on their associated CBM modules. For instance, the processivity of processive endoglucanases from the GH9 family depended on the presence of the CBM3c module [10, 11]. Similarly, CBM domain also aids the processive movement in some family 5 (GH5) endoglucanases. For example, EG1 from *V. volvariella* had a CBM1 module and a family 5 catalytic domain, and the deletion of CBM1 significantly decreased its processivity [13]. However, for other family 5 endoglucanases, the processivity capability was dependent solely on the catalytic domain [6, 14, 15]. As an illustrative example, three GH5 processive endoglucanases containing different CBM6 modules from *S. degradans* were expressed and characterized. These CBM6 modules only promoted strong binding of the polysaccharide chains but were not necessary for processivity [6]. To date, only two native GH5 processive endoglucanases, Cel5A from *G. trabeum* and CHU_2103 from *C. hutchinsonii*, lacking a CBM domain have been reported [14, 15]. Similarly, the EG5C-1 presented here did not contain any CBM domain and exhibited strong processive ability, whereas the holoenzyme EG5C acted more like a typical endoglucanase. To our best knowledge, only a few reports described the additional CBM reduced the processivity of enzymes. Zheng et al. compared native EG1 with its derivatives containing additional CBMs in terms of catalytic activity and processivity, then observed that additional CBM inserted on either side of the catalytic domain had an adverse effect on activity of processivity of EG1 [13]. The adverse effects of extra CBMs may be due to steric hindrance, whereby multiple CBM domains physically blocked access of cellulose chains to the active site, or preventing the smooth sliding movement of enzyme on cellulose chains [13, 35, 36].

### Cellulose binding properties of EG5C and its derivatives

**Fig. 5:**
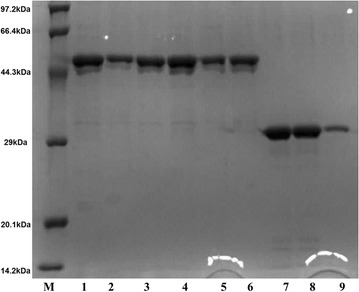
The binding ability of EG5C, EG5C-1 and EG5C-2 for Avicel. Lane M-marker, Lines 1–3-initial, unbound and bound proteins of EG5C, Lines 4–6-initial, unbound and bound proteins of EG5C-2, Lines 7–9-initial, unbound and bound proteins of EG5C-1. Bound proteins of EG5C and its derivatives were released from Avicel using the sample buffer of SDS-PAGE

The degradation of insoluble cellulosic material by cellulases is thought to involve an initial adsorption of the enzyme onto the cellulose surface, followed by the formation of a protein-cellulose complex. Therefore, the binding ability of EG5C, EG5C-1 and EG5C-2 to Avicel was examined (Fig. 5). As expected, EG5C and EG5C-2, both containing CBM3a, strongly bound to Avicel because the majority of these enzymes were observed in the bound fractions (Fig. 5, lines 1–6). In contrast, EG5C-1 bound weaker towards Avicel as most of the protein remained in the unbound fraction (Fig. 5, lines 7–9). Nonetheless, even with EG5C-1, a substantial fraction remained bound to Avicel, contrary to previous studies with other processive endoglucanases, where the catalytic domain alone had no binding affinity to Avicel [10, 21, 23, 28]. The other known exception is the family 5 processive endoglucanase CHU_2103 from *C. hutchinsonii* [15]. To date, the reduction of the costs associated with the production of cellulases is an important prerequisite for the successful commercialization of biomass saccharification. One potential strategy to achieve cost reduction is the recycling of the enzymes. Thus, a major advantage of CBM-lacking cellulase such as EG5C-1 is that a significantly higher fraction of non-bound but active enzyme can be recovered after the hydrolysis, when compared to counterparts that contain a CBM domain [8, 34]. In this regard, the processive endoglucanase EG5C-1, which exhibited high specific activity and weak binding capacity, is a great candidate for potential industrial applications.

### Enzymatic action mode of EG5C and EG5C-1

**Fig. 6:**
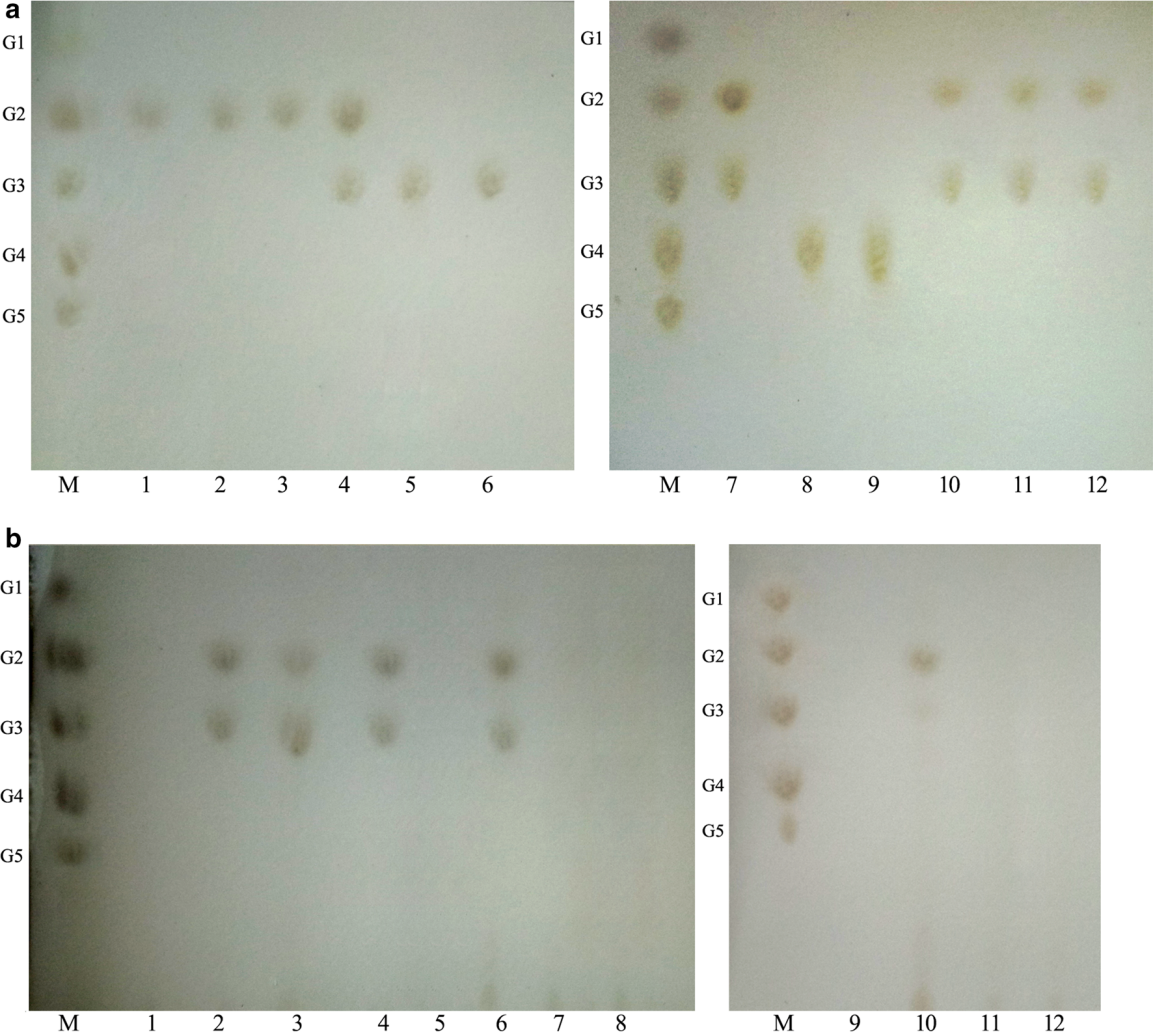
TLC analysis of the hydrolysis products from various cellulosic substrates by EG5C and its derivatives. **a** Hydrolysis products from cello-oligosaccharides. Lane M-glucose unit markers, i.e. glucose (G1), cellobiose (G2), cellotriose (G3), cellotetraose (G4) and cellopentaose (G5), Lanes 1–3-products released from cellobiose by EG5C-1, EG5C and EG5C-2, Lanes 4–6-products released from cellotriose by EG5C-1, EG5C and EG5C-2, Lanes 7–9-products released from cellotetraose by EG5C-1, EG5C and EG5C-2, Lanes 10–12-products released from cellopentaose by EG5C-1, EG5C and EG5C-2. **b** Products obtained from the hydrolysis of CMC, PASC and Avicel. Lane-M glucose unit markers, i.e. glucose (G1), cellobiose (G2), cellotriose (G3), cellotetraose (G4) and cellopentaose (G5), Lanes 2–4-hydrolysis products from CMC by EG5C-1, EG5C and EG5C-2, Lanes 6–8-hydrolysis products from PASC by EG5C-1, EG5C and EG5C-2, Lanes 10–12-hydrolysis products from Avicel by EG5C-1, EG5C and EG5C-2, Lanes 1, 5, 9-supernatant of CMC, PASC and Avicel in the absence of enzyme

TLC was performed to analyze the hydrolysis products of cellulosic substrates treated with EG5C and EG5C-1. For cello-oligosaccharides, while EG5C-1 had no detectable activity towards cellobiose, it cleaved cellotriose (although no glucose was detected). Similarly, EG5C-1 also hydrolyzed cellotetraose and cellopentaose with cellobiose and cellotriose being the main products formed (Fig. 6a). However, the native EG5C and the EG5C-2 variant exhibited different cleavage pattern when compared to EG5C-1. These forms hydrolyzed cellopentaose, producing cellobiose and cellotriose, but did not cleave cellotriose and cellotetraose (Fig. 6a). EG5C-1 was thus one of only a few known processive endoglucanases (also including CHU_2103, EngZ and Cle9 [15, 36, 37]) that could hydrolyze small substrates, while the majority of enzymes in this group (e.g. ChCel5A from *C. hutchinsonii*, Cel9B from *P. barcinonensis*, Cel5 from *H. chejuensis* KCTC 2396 and EG1 from *V. volvacea* [5, 10, 13, 21]), only operated on cello-oligosaccharides with a polymerization degree > 3. The observation that only cellobiose was detected upon the hydrolysis of cellotriose by EG5C-1 (Fig. 6a) indicated this variant might have transglycosylation activity. During cellulose degradation, transglycosylation action would reduce the level of glucose, which in turn might decrease the inhibitory effect of glucose on other cellulolytic enzymes, such as some of β-glucosidases [15]. While some retaining endoglucanases had also been reported to display transglycosylation activity, most of them were typical endoglucanases [15]. To the best of our knowledge, apart from EG5C-1, only CHU_2103 and MtEG5 have been reported to be a processive endoglucanase with transglycosylation activity [7, 15].

Unlike classical endoglucanases that randomly cleave cellulose polymers to form a variety of degradation products, EG5C-1 released predominantly cellobiose and cellotriose as end products when hydrolyzing the insoluble cellulose substrates PASC and Avicel (Fig. 6b), while the substrate CMC was cleaved into a mixture of cellobiose, cellotriose and cellotetraose. As expected, when Avicel and PASC were hydrolyzed by EG5C, only trace amounts of cellobiose and cellotriose were detectable. Nevertheless, for most processive endoglucanases, cellobiose was the major product originating from the hydrolysis of insoluble cellulose. EG5C-1 differed from these enzymes most likely due to its transglycosylation activity.

### Hydrolysis of filter paper and PASC by EG5C-1

The capacity of EG5C and EG5C-1 to solubilize Whatman NO.1 filter paper was demonstrated in Additional file 1: Fig. S4A. In the presence of EG5C-1, the solution turned turbid within 24 h of incubation, indicating that EG5C-1 broke down filter paper efficiently, producing fine fibers. In contrast, after a similar incubation with EG5C, most of the filter paper appeared nearly intact. An essential feature for the efficient degradation of filter paper is the synergistic effect from the combined action of endo- and exoglucanases [29]. The observations that EG5C-1 was rather efficient in solubilizing filter paper on its own, further supported its role as a bifunctional enzyme possessing both endo- and exoglucanase activities. Similarly, efficient degradation of filter paper by family 9 processive endoglucanase CelI and Cle9 was also reported in previous studies [37, 38]. An analysis using TLC has revealed that most of the soluble product released by EG5C-1 was cellobiose and cellotriose (Additional file 1: Fig. S4B).

**Fig. 7:**
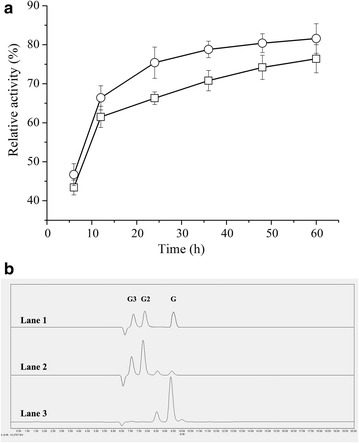
**a** Hydrolysis yield of PASC by EG5C-1 alone (□), or EG5C-1 in combination with Novozyme 188 (○). This experiment was carried out at pH 6.5 in Na_2_HPO_4_/KH_2_PO_4_ buffer at 40 °C with 5% PASC. The EG5C-1 loading was 10 FPU/g PASC. The loading for Novozyme 188 was 20 U/g PASC. Error bars present standard deviations based on three independent experiments. **b** HPLC analysis of the products from PASC hydrolysis by EGC5-1 alone (lane 2), or by a combination of EG5C-1 and Novozyme 188 (lane 3) after 60 h of incubation. Lane 1-standards of G1 to G3

The hydrolysis of PASC was monitored either in presence of only EG5C-1 or in presence of a combination of EG5C-1 and the commercial β-glucosidase Novozyme 188 (an enzyme that degrades predominantly cellobiose) to evaluate saccharification capacity of EG5C-1 on PASC and its potential synergy with β-glucosidase (Fig. 7a). The resulting hydrolytic products were analyzed by HPLC (Fig. 7b). The initial hydrolysis study showed that the glucan conversion reached 76.5% after 60 h of hydrolysis with EG5C-1 alone at the 10 FPU/g PASC enzyme loading. Supplement Novozyme 188 with the dose of 20 U/g PASC, after 60 h of reaction, the conversion yield was slightly increased to 81.6%. On the other hand, HPLC analysis showed that cellobiose and cellotriose were the dominant hydrolytic products from PASC using EG5C-1 alone. As expected, glucose was the main product found in combination action of EG5C-1 and the β-glucosidase. It was well known that cellobiose was the main product of typical exoglucanases. However, typical exoglucanases were very sensitive to cellobiose inhibition [7]. Our observation suggested that in contrast with exoglucanases, the cellobiose inhibition of EG5C-1 was much weaker. Similar results were also reported by Karnaouri et al. [7]. Taken together, EG5C-1 was a very promising candidate catalyst for the conversion of lignocellulosic biomass.

### Key amino acid residues for processivity of EG5C-1

**Fig. 8:**
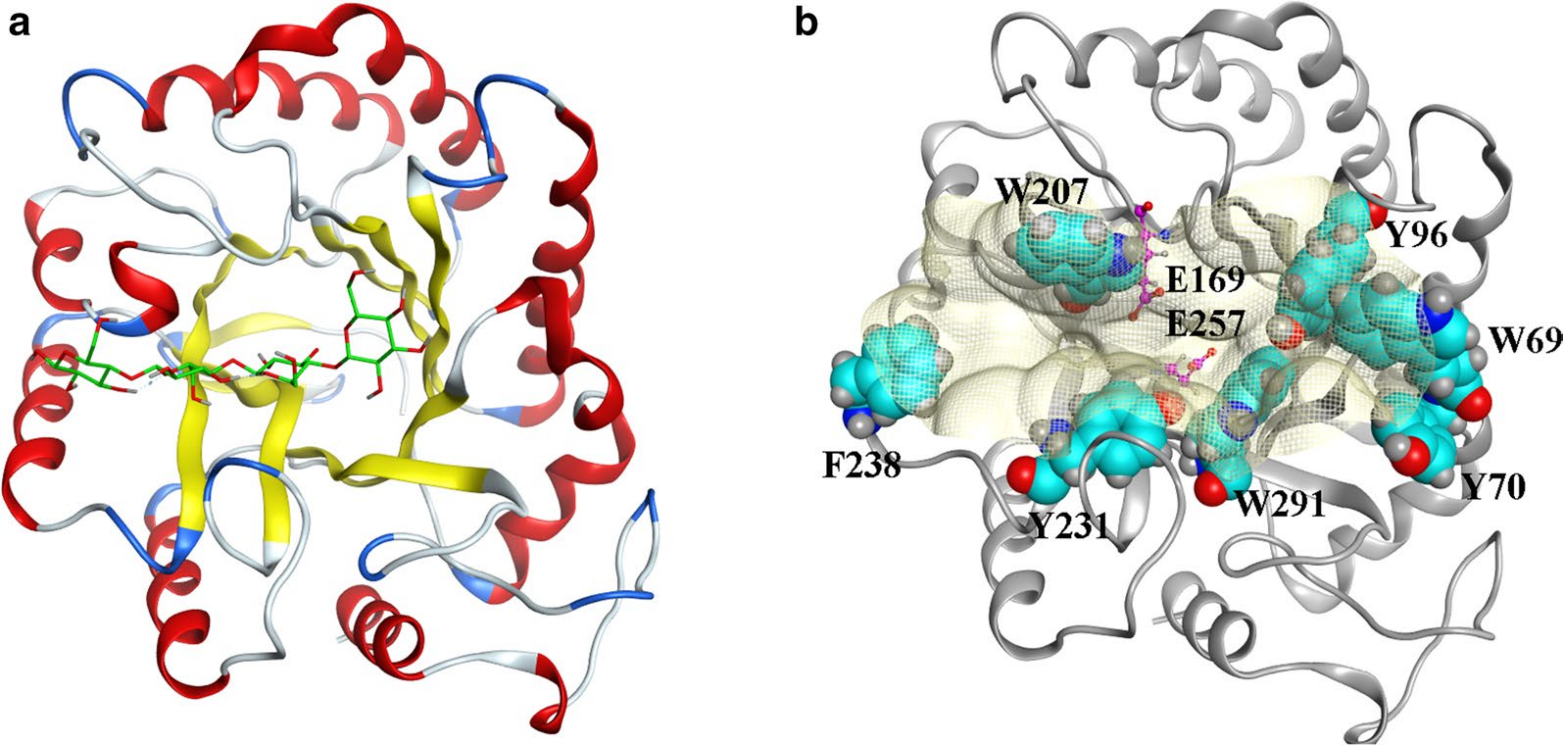
Model of the three dimensional structure of EG5C-1. **a** The protein structure of EG5C-1 shown as cartoon representations with *β*-strands in yellow, *α*-helices in red, loops in grey and turn in blue, and the docked substrate cellotetraose styled in green stick representation. **b** Surface representation of the binding pocket in EG5C-1, highlighting the seven aromatic residues Trp69, Tyr70, Tyr96, Trp207, Tyr231, Phe238 and Trp291 shown as cyan spheres, and the catalytic residues Glu169 and Glu257, shown as violet sticks

In bacterial systems, the majority of processive endoglucanases belong to the GH9 family [6]. Insofar, the processive capability of the GH5-family EG5C-1 is unusual. A phylogenetic analysis also revealed that the primary sequence of EG5C-1 was sufficiently divergent from the majority of other family 5 processive endoglucanases (Additional file 1: Fig. S3). The endoglucanase that shared the highest degree of sequence identity with EG5C-1 (99%) and for which a crystal structure was available was the enzyme BsCel5A from *B. subtilis* 168 [18]. Based on its structure, a homology model for EG5C-1 structure was established. EG5C-1 had a classic (β/α)_8_-barrel fold and a deep substrate-binding cleft formed by the C-terminal ends of β-strands (Fig. 8a). It has been proposed that a long tunnel or deep cleft is essential for processivity [35, 39]. Furthermore, a common means was accomplished by the aromatic amino acids lining the substrate-binding pocket, wherein aromatic rings could stack with planar faces of carbohydrate rings via carbohydrate-π interactions [40–43]. In this context, the function of aromatic residues in enzyme tunnels/clefts was not only to facilitate binding affinity but also to influence processive ability on crystalline or amorphous substrates.

**Fig. 9:**
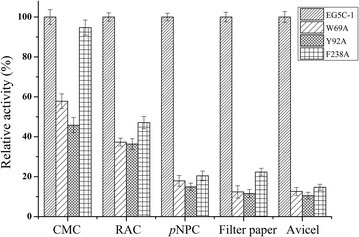
Effect of site-directed mutations of selected aromatic residues of EG5C-1 on the hydrolysis activity towards CMC, PASC, *p*NPC, filter paper and Avicel. Error bars are standard deviation of 3 independent experiments

In this study, there were seven aromatic amino acid residues, W69, Y70, Y96, W207, Y231, F238 and W291, distributed in the binding cleft of enzyme EG5C-1 (Fig. 8b). W69 and Y70 were positioned at the entrance to the cleft, while F238 was at other end of the canyon. Y96 was located at the bottom of the cleft, while W207 was lining the wall of the cleft and was located close to catalytic residue E169. Y231 and W291 also lied at the bottom of the pocket, and near another catalytic residue, E257. In order to evaluate the roles of these residues in the catalytic mechanism and processivity of EG5C-1, these aromatic aminos were replaced by alanine. The mutations of Y70, W207, Y231 and W291 resulted in the complete loss of the hydrolytic activity regardless of the substrates used. The W69A and Y96A mutants also had a decreased activity towards a range of substrates (Fig. 9). Interestingly, the F238A mutant retained more than 90% of the specific activity of the native enzyme against CMC. In contrast, a dramatically decreased hydrolytic activity toward PASC, *p*NPC, filter paper and Avicel was observed (Fig. 9). Processivity of F238A mutant was markedly decreased with a ratio of soluble to insoluble reducing ends of about 2.0–2.6 as the incubation time was prolonged from 15 min to 180 min (Fig. 4). F238 thus appeared to play an essential role for the effective hydrolysis of EG5C-1 towards insoluble amorphous or crystalline cellulose, while having only a minor effect on CMCase activity. Moreover, this residue was essential to the processive activity of EG5C-1. Upon its replacement by alanine, the substrate preference of the mutant was similar to that of typical endoglucanases. Finally, the cellulose binding ability of the mutant enzymes was measured. Although substitutions of most of aromatic amino acid residues led to loss of their hydrolytic activity, the adsorption capacity of these mutants to cellulose unchanged, indicating that the loss in activity was not due to disruption of the substrate binding site. An interesting mutation was F238A, which almost completely bound to cellulose (Additional file 1: Table S3). In this context, an obvious conclusion was reached that the aromatic amino acid F238 played a marked influence not only on the continuous moving of EG5C-1 along the cellulose chain, but also on cellulose binding.

To date, experimental and computational investigations indicated that aromatic residues at the entrance and exit of exoglucanase played an important role in binding, processivity and stabilization of carbohydrate ligands [40, 41, 44]. In addition, two GH18 family chitinases ChiA and ChiB, which did not require a CBM for their processivity, were reported. The study indicated that these two enzymes have deep substrate binding clefts lined with aromatic residues. Further, the contribution of aromatic residues to the processivity of ChiA and ChiB was studied. In ChiA, the W167 in the − 3 subsite seemed crucial for processivity. In ChiB, W97 in the + 1 subsite and W220 in the + 2 subsite were crucial for processivity [45]. However, for GH5 processive endoglucanases, until now only a few works on the mechanism of processivity of catalytic domain has been reported. Zhu et al. reported that substitutions of aromatic amino acids including W61 and W308 at the entrance of catalytic cleft of ChCle5A dramatically decreased hydrolytic activity toward filter paper while causing only a slight decrease in CMCase [5]. However, the changes in processivity between ChCle5A and mutant enzymes were not detected. Afterwards, Zhang et al. mutated a tryptophan W197 in a family 5 processive endoglucanase CHU_2103 from *C. hutchinsonii* to alanine. The mutated enzyme still retained nearly 80% specific activity on CMC and PASC compared with wild enzyme, but the processivity was markedly decreased. Moreover, this mutant almost completely lost the cellulose binding activity [15]. In contrast, in our studies, although the F238A mutant enzyme of EG5C-1 also exhibited the decreased processivity, its cellulose binding ability was markedly improved.

## Conclusions

In the present study, the endoglucanase EG5C and its truncated form EG5C-1 from *B. subtilis* BS-5 were expressed in *E. coli,* purified and characterized for their potential in biomass conversion. EG5C was a typical endoglucanase consisting of a family 5 catalytic domain and a CBM3 domain, and was only an efficient catalyst for the hydrolysis of soluble cellulosic substrates. However, EG5C-1 exhibited high activities for soluble CMC as well as insoluble amorphous and crystalline cellulose substrates, indicating that EG5C-1 was a processive endoglucanase. Moreover, EG5C-1 alone was capable to efficiently hydrolyze filter paper and PASC, releasing cellobiose as the main product. Site-directed mutagenesis demonstrated that the aromatic amino acid F238 at the entrance of the substrate binding cleft played an important role not only for processive activity, but also for cellulose binding ability. Our combined data demonstrate that EG5C-1 has strong industrial potential for the degradation of cellulosic biomass for biofuel production. In addition, due to the distinct functional differences of EG5C, EG5C-1 and the mutant F238, these systems provide an excellent background to investigate the molecular-level details for the mechanism of action of a poorly studied group of enzymes. Efforts towards these goals are in progress.

## Additional file

**Additional file 1: Figure S1.** **A** The internal fragments of EG5C and EG5C-1 show significant homology to those of the endoglucanase from *B. subtilis* 168. Matched peptides of EG5C with those of endoglucanase from *B. subtilis* 168 are underlined with solid lines. The dotted lines indicate matched peptides of EG5C-1 with those of endoglucanase from *B. subtilis* 168. **B** Schematic structures of EG5C and its derivates. **Figure S2.** Multiple sequence alignment between EG5C and other family 5 endoglucanases from *B. subtilis* 168, *B. subtilis* A53, *B. subtilis* UMCT and *B. amyoliquefaciens* DL-3. **Figure S3.** Multiple sequence alignment of EG5C-1 and other family 5 processive endoglucanases or their catalytic domains. The following sequences have been included: catalytic domain of Cel5H from *S. degradans*, catalytic domain of ChCel5A from *C. hutchinsonii*, catalytic domain of Cel5C from *H. chejuensis* KCTC 2396, catalytic domain of Cel5A from uncultured Bacterium, catalytic domain of MtEG5A from *M. thermophile*, enzyme CHU_2103 from *C. hutchinsonii*, enzyme Cel5A from *G. trabeum*. CHU_2103 from *C. hutchinsonii* and Cel5A from *G. trabeum* were GH5 processive endoglucanase without CBM. **Figure S4. A** Solubilization of filter paper by EG5C and EG5C-1. **B** TLC analysis of soluble sugars following the hydrolysis of filter paper by EG5C and EG5C-1. Lane M – glucose unit markers, i.e. glucose (G1), cellobiose (G2), cellotriose (G3), cellotetraose (G4) and cellopentaose (G5). **Table S1.** Primers used for plasmid construction of EG5C, EG5C-1 and EG5C-2. **Table S2.** Primers used for the generation of site-directed mutants of EG5C-1. **Table S3.** Cellulose binding properties of EG5C-1 and its site-directed mutants.

## Abbreviations

CMC: carboxymethyl cellulose
PASC: phosphoric acid-swollen cellulose
*p*-NPC: *p*-nitrophenyl-β-d-cellobioside
FP: filter paper
GH: glycoside hydrolase
CBM: carbohydrate binding module
LC–MS/MS: liquid chromatography-tandem mass spectrometry
LB medium: Luria-Bertani medium
SDS-PAGE: sodium dodecyl sulfate polyacrylamide gel electrophoresis
TLC: thin-layer chromatograph
HPLC: high performance liquid chromatography
DNS: dinitrosalicylic acid
