# Supplementary material for: Processivity and enzymatic mechanism of a multifunctional family 5 endoglucanase from Bacillus subtilis BS-5 with potential applications in the saccharification of cellulosic substrates
Source: Biotechnol Biofuels. 2018 Jan 29;11:20. doi: 10.1186/s13068-018-1022-2 (PMC5787917; doi:10.1186/s13068-018-1022-2)
Supplement: Supplementary file 1 — Additional file 1: Figure S1. A The internal fragments of EG5C and EG5C-1 show significant homology to those of the endoglucanase from B. subtilis 168. Matched peptides of EG5C with those of endoglucanase from B. subtilis 168 are underlined with solid lines. The dotted lines indicate matched peptides of EG5C-1 with those of endoglucanase from B. subtilis 168. B Schematic structures of EG5C and its derivates. Figure S2. Multiple sequence alignment between EG5C and other family 5 endoglucanases from B. subtilis 168, B. subtilis A53, B. subtilis UMCT and B. amyoliquefaciens DL-3. Figure S3. Multiple sequence alignment of EG5C-1 and other family 5 processive endoglucanases or their catalytic domains. The following sequences have been included: catalytic domain of Cel5H from S. degradans, catalytic domain of ChCel5A from C. hutchinsonii, catalytic domain of Cel5C from H. chejuensis KCTC 2396, catalytic domain of Cel5A from uncultured Bacterium, catalytic domain of MtEG5A from M. thermophile, enzyme CHU_2103 from C. hutchinsonii, enzyme Cel5A from G. trabeum. CHU_2103 from C. hutchinsonii and Cel5A from G. trabeum were GH5 processive endoglucanase without CBM. Figure S4. A Solubilization of filter paper by EG5C and EG5C-1. B TLC analysis of soluble sugars following the hydrolysis of filter paper by EG5C and EG5C-1. Lane M – glucose unit markers, i.e. glucose (G1), cellobiose (G2), cellotriose (G3), cellotetraose (G4) and cellopentaose (G5). Table S1. Primers used for plasmid construction of EG5C, EG5C-1 and EG5C-2. Table S2. Primers used for the generation of site-directed mutants of EG5C-1. Table S3. Cellulose binding properties of EG5C-1 and its site-directed mutants. [file 13068_2018_1022_MOESM1_ESM.doc]

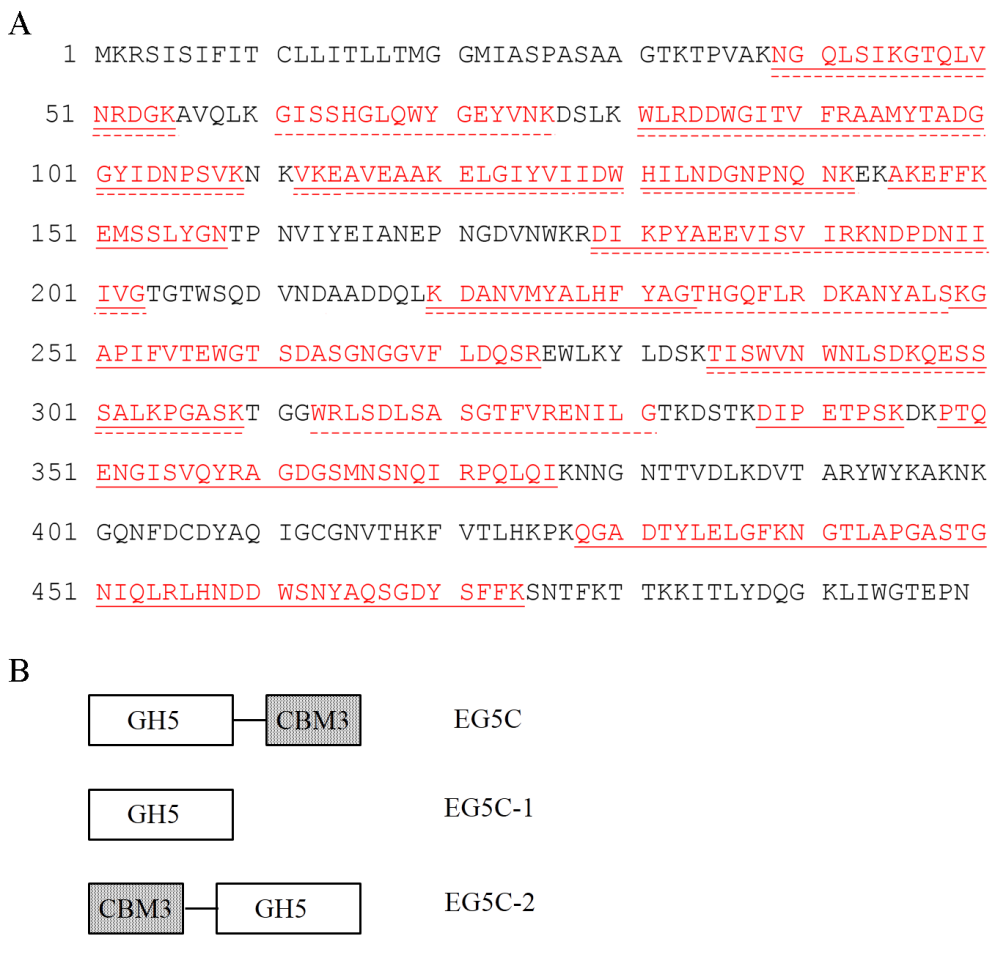


**Fig S1 A** The internal fragments of EG5C and EG5C-1 show significant homology to those of the endoglucanase from *B. subtilis* 168. Matched peptides of EG5C with those of endoglucanase from *B. subtilis* 168 are underlined with solid lines. The dotted lines indicate matched peptides of EG5C-1 with those of endoglucanase from *B. subtilis* 168. **B** Schematic structures of EG5C and its derivates.


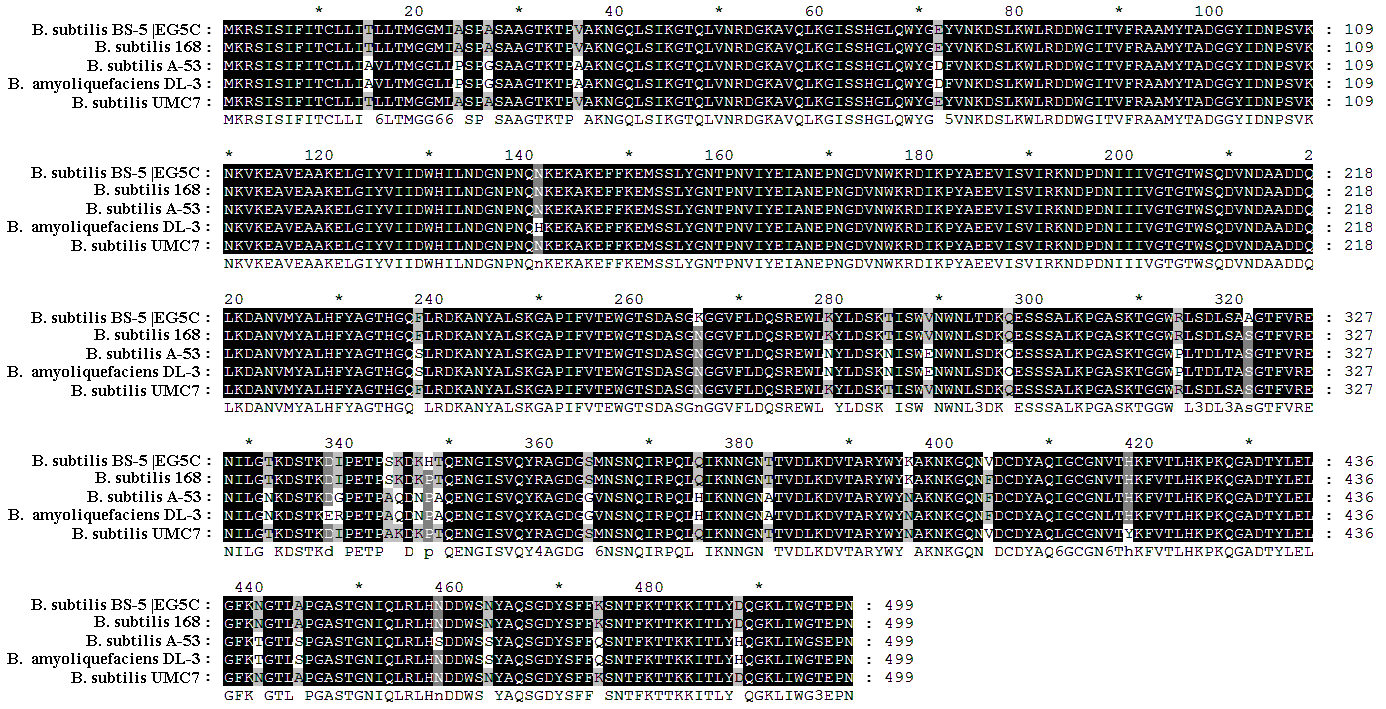


**Fig. S2** Multiple sequence alignment between EG5C and other family 5 endoglucanases from *B. subtilis* 168, *B. subtilis* A53, *B. subtilis* UMCT and *B. amyoliquefaciens* DL-3.

**
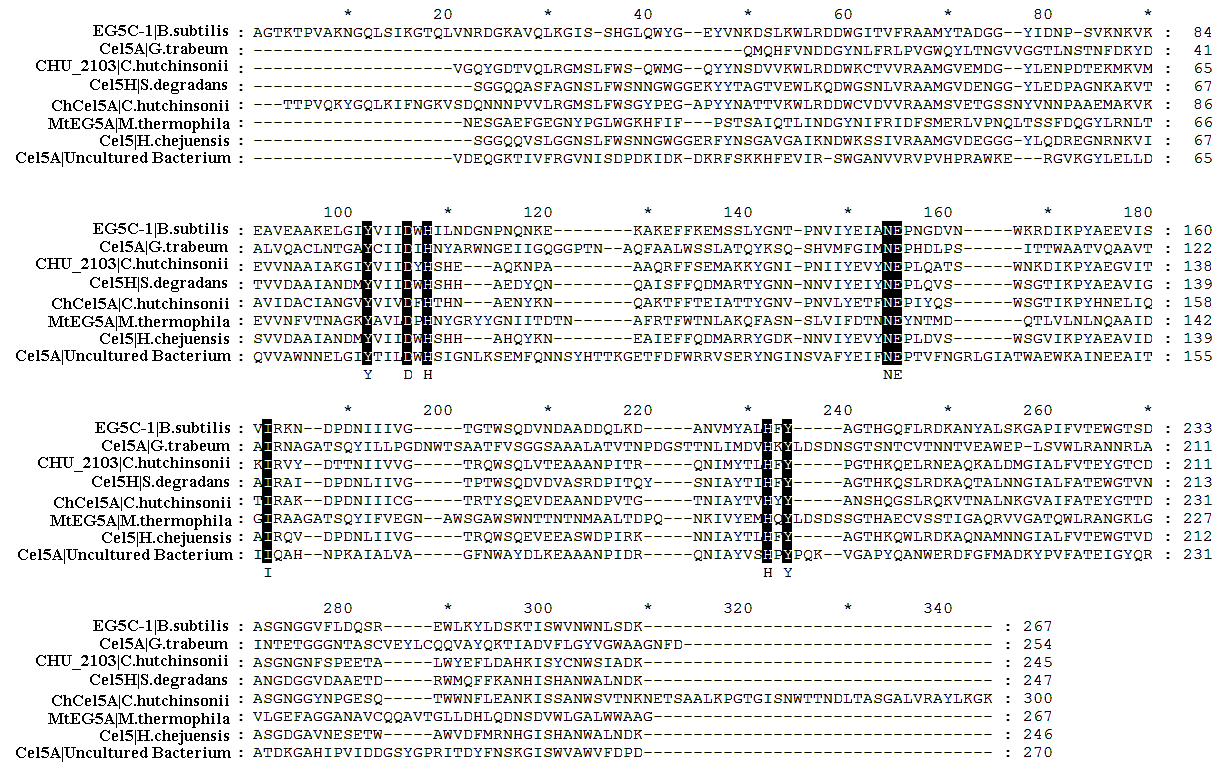
**

**Fig. S3** Multiple sequence alignment of EG5C-1 and other family 5 processive endoglucanases or their catalytic domains. The following sequences have been included: catalytic domain of Cel5H from *S. degradans*, catalytic domain of ChCel5A from *C. hutchinsonii*, catalytic domain of Cel5C from *H. chejuensis* KCTC 2396, catalytic domain of Cel5A from uncultured Bacterium, catalytic domain of MtEG5A from *M. thermophile*, enzyme CHU_2103 from *C. hutchinsonii*, enzyme Cel5A from *G. trabeum*. CHU_2103 from *C. hutchinsonii* and Cel5A from *G. trabeum* were GH5 processive endoglucanase without CBM.


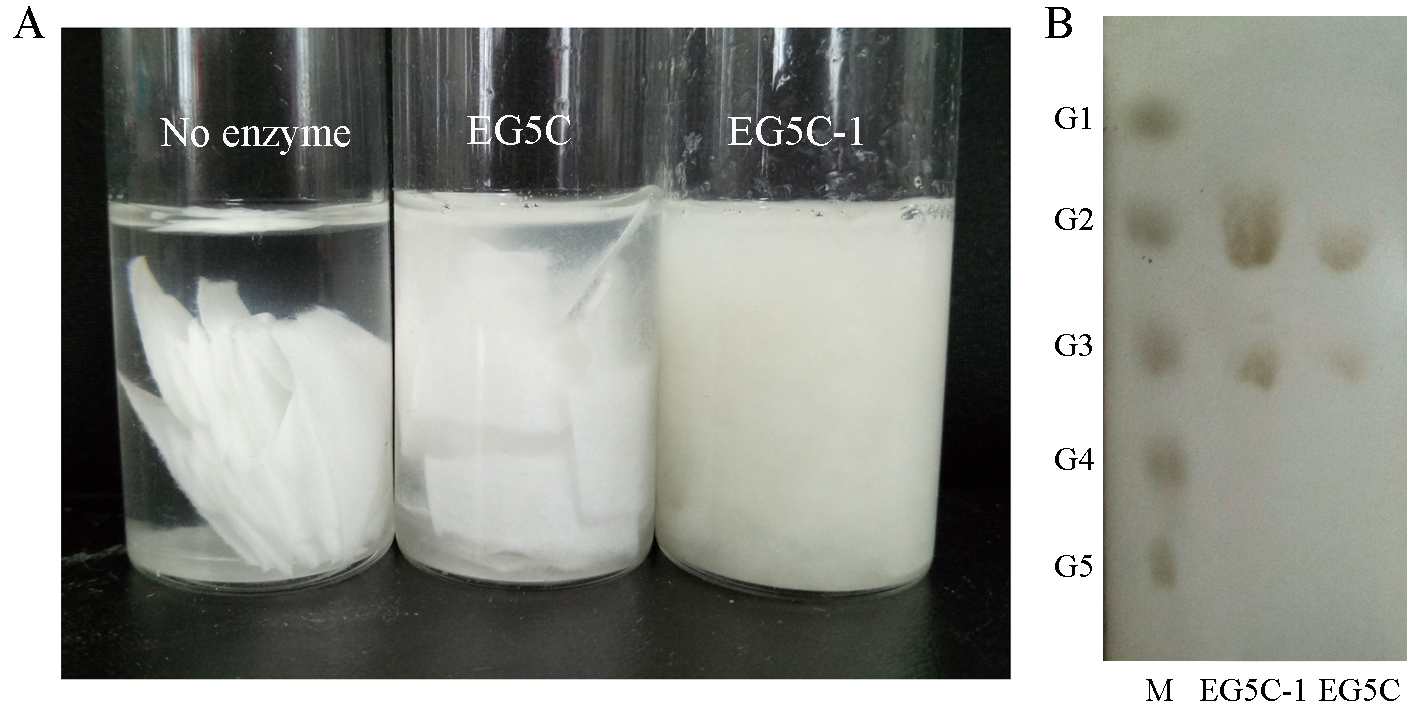


**Fig. S4 A** Solubilization of filter paper by EG5C and EG5C-1. **B** TLC analysis of soluble sugars following the hydrolysis of filter paper by EG5C and EG5C-1. Lane M – glucose unit markers, *i.e.* glucose (G1), cellobiose (G2), cellotriose (G3), cellotetraose (G4) and cellopentaose (G5).

Table S1 Primers used for plasmid construction of EG5C, EG5C-1 and EG5C-2

| Primers | Sequences (5’ to 3’) |
| --- | --- |
| EG5C-F | GCCGCCATGGGCGCAGGGACAAAAACGCC (*Nco*Ⅰ) |
| EG5C-R | CCGCTCGAGCTAATTTGGTTCTGTTCCC (*Xho*Ⅰ) |
| EG5C-1-F | GCCGCCATGGGCGCAGGGACAAAAACGCCAG (*Nco*Ⅰ) |
| EG5C-1-R | CCGCTCGAGGCCGAGAATGTTTTCTCTAACG (*Xho*Ⅰ) |
| EG5C-2-F | GGCCATGGCGATTTCTGTACAGTACAGAGCAG (*Nco*Ⅰ) |
| EG5C-2-CBM-R | CCTTCGTCGAATCTTTGGTCTAATTTGGTTCTGTTCCC |
| EG5C-2-LIN-F | ACCAAAGATTCGACGAAGGAC |
| EG5C-2-LIN-R | ACCATTTTCCTGTGTGTGTTTATC |
| EG5C-2-GH-F | CACACACAGGAAAATGGTGCAGGGACAAAAACGCC |
| EG5C-2-R | GGCTCGAGGCCGAGAATGTTTTCTCTAACG (*Xho*Ⅰ) |

Table S2 Primers used for the generation of site-directed mutants of EG5C-1.

| Primers | Sequences (5’ to 3’) |
| --- | --- |
| EG5C-1 W69A-F | ctttattgacatattctccatacgcttgcaatccgtgtgaactgatcc |
| EG5C-1 W69A-R | ggatcagttcacacggattgcaagcgtatggagaatatgtcaataaag |
| EG5C-1 Y70A-F | gtctttattgacatattctccagcccattgcaatccgtgtgaactg |
| EG5C-1 Y70A-R | cagttcacacggattgcaatgggctggagaatatgtcaataaagac |
| EG5C-1 Y96A-F | ccgccatctgccgtagccatcgctgcacggaa |
| EG5C-1 Y96A-R | ttccgtgcagcgatggctacggcagatggcgg |
| EG5C-1 W207A-F | CATCCTGGCTCCATGCACCGGTTCCGACAAT |
| EG5C-1 W207A-R | ATTGTCGGAACCGGTGCATGGAGCCAGGATG |
| EG5C-1 Y231A-F | cgtgtgtgccggcagcaaaatgaagtgcgtacataacgtttgc |
| EG5C-1 Y231A-R | gcaaacgttatgtacgcacttcattttgctgccggcacacacg |
| EG5C-1 F238A-F | gccggcacacacggccaagctttacgggataaagcaaa |
| EG5C-1 F238A-R | tttgctttatcccgtaaagcttggccgtgtgtgccggc |
| EG5C-1 W291A-F | ctgcttatcagtaagattcgcgttcacccagctaatggtc |
| EG5C-1 W291A-R | gaccattagctgggtgaacgcgaatcttactgataagcag |

Table S3 Cellulose binding properties of EG5C-1 and its site-directed mutants.

| Enzyme | Binding capacity (%) |
| --- | --- |
| EG5C-1 | 11.8 |
| W69A | 12.3 |
| Y70A | 10.7 |
| Y96A | 9.8 |
| W207A | 8.9 |
| Y231A | 10.4 |
| F238A | 90.4 |
| W291A | 10.1 |
